# Dynamic Assessment of Tissue and Plasma *EGFR*-Activating and T790M Mutations with Droplet Digital PCR Assays for Monitoring Response and Resistance in Non-Small Cell Lung Cancers Treated with EGFR-TKIs

**DOI:** 10.3390/ijms231911353

**Published:** 2022-09-26

**Authors:** Hsiang-Ling Ho, Fang-Yu Wang, Chi-Lu Chiang, Chun-Ming Tsai, Chao-Hua Chiu, Teh-Ying Chou

**Affiliations:** 1 Department of Pathology and Laboratory Medicine, Taipei Veterans General Hospital, Taipei 112201, Taiwan; 2 Department of Biotechnology and Laboratory Science in Medicine, National Yang Ming Chiao Tung University, Taipei 112304, Taiwan; 3 Department of Chest Medicine, Taipei Veterans General Hospital, Taipei 112201, Taiwan; 4 Institute of Clinical Medicine, National Yang Ming Chiao Tung University, Taipei 112304, Taiwan; 5 Department of Oncology, Taipei Veterans General Hospital, Taipei 112201, Taiwan; 6 Taipei Cancer Center and Taipei Medical University Hospital, Taipei Medical University, Taipei 110301, Taiwan; 7 Institute of Biochemistry and Molecular Biology, National Yang Ming Chiao Tung University, Taipei 112304, Taiwan

**Keywords:** *EGFR* mutation, droplet digital PCR assays, EGFR-TKIs, circulating cell-free DNA, resistance mechanisms

## Abstract

Assessing tumor *EGFR* mutation status is necessary for the proper management of patients with advanced non–small cell lung cancer (NSCLC). We evaluated the impact of dynamic analyses of the plasma and tissue *EGFR* mutation using ultra-sensitive droplet digital PCR (ddPCR) assays to manage NSCLC patients treated with EGFR tyrosine kinase inhibitors (EGFR-TKIs). Paired tumor tissues and plasma samples from 137 EGFR-mutated lung adenocarcinoma patients prior to the first-line EGFR-TKIs treatment (at baseline) and at disease progression were subjected to *EGFR* mutation analysis using ddPCR, together with the analyses of the clinicopathological characteristics and treatment outcomes. Patients with *EGFR*-activating mutations detected in baseline plasma were associated with bone metastasis (*p* = 0.002) and had shorter progression-free survival (12.9 vs. 17.7 months, *p* = 0.02) and overall survival (24.0 vs. 39.4 months, *p* = 0.02) compared to those without. Pre-treatment *EGFR* T790M mutation found in baseline tumor tissues of 28 patients (20.4%; 28/137) was significantly associated with brain metastasis (*p* = 0.005) and a shorter brain metastasis-free survival (*p* = 0.001). The presence of *EGFR* T790M mutations in baseline tumor tissues did not correlate with the emergence of acquired *EGFR* T790M mutations detected at progression. At disease progression, acquired *EGFR* T790M mutations were detected in 26.6% (21/79) of the plasma samples and 42.9% (15/35) of the rebiopsy tissues, with a concordance rate of 71.4% (25/35). The dynamic monitoring of tissue and plasma *EGFR* mutation status at baseline and progression using ddPCR has a clinical impact on the evaluation of EGFR-TKIs treatment efficacy and patient outcomes, as well as the emergence of resistance in NSCLC.

## 1. Introduction

Lung cancer is the leading cause of cancer-related death around the world. Nearly 85% of lung cancer patients have non–small cell lung cancer (NSCLC), with an overall 5-year survival rate of less than 15% [1,2]. Epidermal growth factor receptor (EGFR) mutations are the most frequent oncogenic driver mutations found in NSCLC. Mutations of the *EGFR* gene that occur in exons 18 to 21 within the kinase domain, leading to the activation of the kinase activity by a ligand-independent mechanism, are referred to as “activating mutations”. *EGFR*-activating mutations are highly associated with tumor responses to EGFR-TKIs. *EGFR* exon 19 deletions and L858R mutation are the two most common activating mutations in NSCLC [3]. More than 50% of Asian NSCLC patients were found to harbor *EGFR*-activating mutations, which are more prevalent in female and never-smokers [4]. EGFR tyrosine kinase inhibitors (EGFR-TKIs) treatment, demonstrating superior progression-free survival, higher tumor response rates and a better quality of life, are now recommended as the primary therapy for advanced-stage NSCLC patients with *EGFR*-activating mutations [5,6,7,8,9]. However, most patients who initially respond to first- or second-generation EGFR-TKIs will eventually undergo disease progression after a median of 9 to 14 months due to acquired resistance [6,7,8,10,11]. Approximately 50 to 60% of cases of acquired resistance for first- and second-generation EGFR-TKIs are due to the emergence of secondary *EGFR* T790M mutation that affects the “gatekeeper residue” in the catalytic domain of the kinase, leading to steric hindrance of EGFR-TKI binding due to the presence of the bulkier methionine side chain in the ATP-kinase-binding pocket and subsequently abolishing the potency of ATP-competitive TKIs [3,12]. Recently, the third-generation EGFR-TKI, osimertinib, targeting both *EGFR*-activating mutations as well as the gatekeeper *EGFR* T790M mutation, has been approved as a first-line treatment for patients with EGFR-mutant metastatic NSCLC and as a second-line therapy for patients who have developed *EGFR* T790M mutation after failure of the first- and/or second-generation EGFR-TKI treatment [13,14]. Therefore, the continuous assessment of tumor *EGFR* mutation status during the course of disease is necessary for the better management of NSCLC patients, particularly for the early identification of the resistance mechanisms.

Currently, the gold standard for assessing *EGFR* mutations is to analyze the tumor tissues. However, obtaining tumor samples through tumor biopsies has certain limitations [15,16]. First, tumor biopsy is an invasive and potentially risky procedure. Second, a small amount of tumor tissue obtained from a single-site biopsy has a high failure rate in molecular testing and is further affected by intra-tumoral heterogeneity and sampling bias [17]. Recently, testing plasma circulating cell-free DNA (cfDNA), commonly known as liquid biopsy, has been highlighted as a promising alternative. Unlike traditional tumor biopsy, liquid biopsy is less invasive and allows for frequent sampling during follow-up. Liquid biopsy contains tumor DNA released from both the primary and/or metastatic tumor sites, which can better reflect the molecular heterogeneity of tumors [18,19].

Given that the tumor tissue is extremely heterogeneous, and the concentration of tumor DNA in peripheral blood is extremely low and varies between patients, molecular methods with a high detection sensitivity are required for more accurate diagnostics. The cobas EGFR Mutation Test is a real-time polymerase chain reaction (PCR) test that can qualitatively identify 42 mutations in exons 18, 19, 20 and 21 of the *EGFR* gene, including the T790M resistance mutation, by using allele-specific PCR primers specifically amplifying the targeted mutant sequences rather than wild-type sequences and/or other human genomic DNA. It has been clinically validated as a companion diagnostic (CDx) for EGFR-TKIs therapy in patients with advanced NSCLC with a detection sensitivity of around 5% for tissue-derived DNA [20]. Droplet digital PCR (ddPCR) is an ultra-sensitive assay combining microfluidics technology with TaqMan-based quantitative PCR (qPCR) to measure absolute mutation alleles through clonal amplification and fluorescence detection of tens of thousands of individual template molecules in a single reaction. Unlike classic qPCR, which depends on calibration curves for target sequence quantification, ddPCR collects fluorescence signals via end-point measurements and utilizes Poisson statistics to calculate the target concentrations, which could avoid the pitfalls associated with the variations in reaction efficiencies. Therefore, ddPCR has emerged as a promising tool for the detection and absolute quantification of gene mutations below 1% [21,22,23,24]. As ddPCR is increasingly implemented in clinical practice for detecting somatic mutations that are present at low frequencies in tumors or circulating cell-free DNA, the current study aimed to evaluate the clinical utility of assessing both plasma and tissue *EGFR* mutations using ultra-sensitive ddPCR assays prior to the treatment and at disease progression in monitoring the efficacies and outcomes of NSCLC patients treated with EGFR-TKIs.

## 2. Results

### 2.1. Patient Characteristics

A total of 137 lung adenocarcinoma patients with *EGFR*-activating mutations identified from baseline tumor biopsies using a routine cobas EGFR Mutation Test were enrolled in this study. The majority of patients (98.5%; 135/137) presented at stage IV, and the rest were at stage IIIB. There were 76 (55.5%; 76/137) females and 61 (44.5%; 61/137) males, with a median age of 64.3 years. Among these patients, 101 (73.7%; 101/137) were never smokers, whereas all others were smokers. According to the baseline tissue testing results, 77 (56.2%; 77/137) patients had L858R mutations, and 60 (43.8%; 60/137) had exon 19 deletions. All these patients received first- or second-generation EGFR-TKIs as their first-line therapy; 74 (54.0%; 74/137) were treated with afatinib, 41 (29.9%; 41/137) were treated with erlotinib and 22 (16.1%; 22/137) were treated with gefitinib. Gefitinib and erlotinib are the first-generation EGFR-TKIs that reversibly bind to EGFR and inhibit EGFR signaling, while afatinib is a second-generation EGFR-TKI that irreversibly blocks signaling from all members of the ErbB receptor family [25]. For distant metastasis, there were 56 (40.9%; 56/137) patients with brain metastases, 71 (51.8%; 71/137) with bone metastases, 34 (24.8%; 34/137) with pleura metastases and 13 (9.5%; 13/137) with liver metastases. Brain metastases were presented in 37 (66.1%; 37/56) of the cases at the time of accrual, bone metastases were present in 45 (63.4%; 45/71), pleura metastases were present in 28 (82.4%; 28/34) and liver metastases were present in 7 (53.8%; 7/13).

### 2.2. Detection of Tissue and Plasma EGFR Mutations at Baseline Using the ddPCR Platform

The baseline tissue and plasma samples were subjected to *EGFR* mutation analysis using the ddPCR platform. The baseline tissue analysis revealed that 61 (44.5%; 61/137) patients were positive for L858R mutations, 16 (11.7%; 16/137) were positive for L858R combined with T790M mutations, 48 (35.0%; 48/137) were positive for exon 19 deletions and 12 (8.8%; 12/137) were positive for exon 19 deletions combined with T790M mutations. The concordance rate between the cobas EGFR Mutation Test and the ddPCR platform for baseline tissue testing was 79.6% (109/137) for overall *EGFR* mutations, including activating and T790M mutations, and it was 100% (137/137) for activating mutations only (Table 1). Remarkably, 20.4% (28/137) of the baseline cases were found to harbor pre-treatment *EGFR* T790M mutation by the ddPCR platform, but none of them were detected by the cobas EGFR Mutation Test. As determined by baseline plasma analysis using ddPCR, 89 (65.0%; 89/137) patients were positive for *EGFR* mutations, including 47 (34.3%; 47/137) positive for L858R, 2 (1.5%; 2/137) positive for L858R combined with T790M mutations, 38 (27.7%; 38/137) positive for exon 19 deletions and 2 (1.5%; 2/137) positive for exon 19 deletions combined with T790M mutations. A total of 2.9% (4/137) of patients had pre-treatment *EGFR* T790M mutation in the baseline plasma samples. The concordance rates between baseline tissue and plasma testing using the ddPCR platform were 51.8% (71/137) for overall *EGFR* mutations and 65.0% (89/137) for activating mutations only (Table 2).

### 2.3. Clinical Characteristics of Patients with Pre-Treatment Tissue EGFR T790M and Plasma EGFR-Activating Mutations

**Figure 1:**
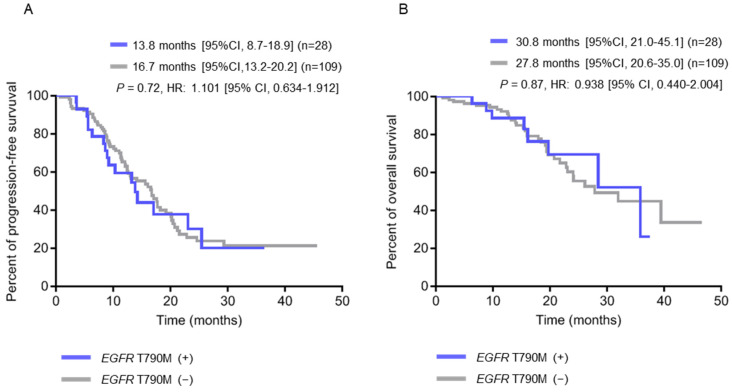
Kaplan-Meier survival analysis according to pre-treatment *EGFR* T790M status in baseline tumor tissue (N = 137). (**A**) Progression-free survival (PFS) curves. (**B**) Overall survival (OS) curves.

To investigate the association between the clinical characteristics of patients and pre-treatment tissue *EGFR* T790M mutation or plasma *EGFR*-activating mutations, Chi-square or Fisher’s exact test was used for analyses, and the results are shown in Table 3. There were no statistical differences for age, gender, smoking history, first-line therapies, primary tumor sizes, TNM stages and *EGFR* mutation types between these two groups. However, we found that patients with pre-treatment *EGFR* T790M mutation were significantly associated with the development of brain metastasis (*p* = 0.005) but not bone, pleura or liver metastasis. To further evaluate if these clinical parameters are independently associated with the mutation status, a multivariate analysis was also performed (Supplemental Table S1). Kaplan–Meier survival analysis was used to estimate the progression-free survival (PFS), overall survival (OS) and brain metastasis-free survival (BMFS). The results showed that the PFS and OS were 13.8 months and 30.8 months, respectively, for patients with pre-treatment *EGFR* T790M mutation and 16.7 months and 27.8 months, respectively, for those without pre-treatment *EGFR* T790M mutation. No significant difference regarding patient survival was found between these two groups (*p* = 0.72 for PFS; *p* = 0.87 for OS) (Figure 1A,B).

**Figure 2:**
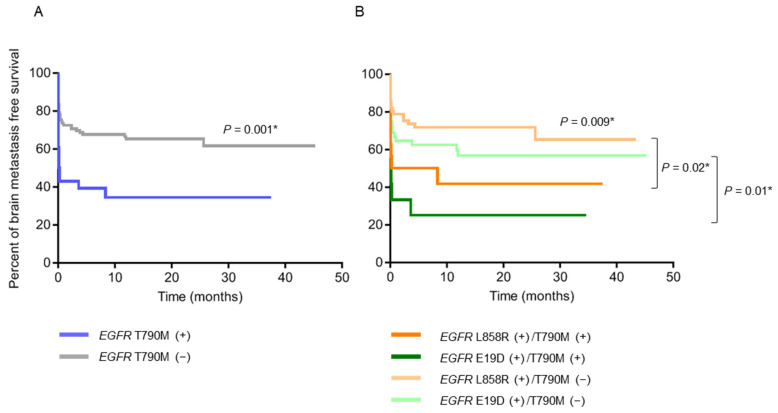
Brain metastasis-free survival (BMFS) analysis (N = 137). (**A**) BMFS curves of patients with pre-treatment *EGFR* T790M (+) (N = 28) and (−) (N = 109). (**B**) BMFS curves of patients with pre-treatment *EGFR* L858R (+)/T790M (+) (N = 16), L858R (+)/T790M (−) (N = 61), E19D (+)/T790M (+) (N = 12) and E19D (+)/T790M (−) (N = 48) in baseline tumor tissue. * *p* < 0.05, indicating significance. Abbreviation: E19D, exon 19 deletions.

**Figure 3:**
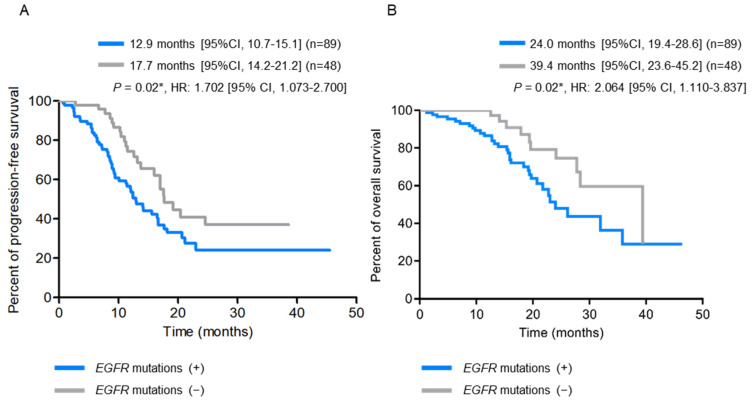
Kaplan-Meier survival analysis according to baseline plasma *EGFR*-activating mutation status (N = 137). (**A**) Progression-free survival (PFS) curves. (**B**) Overall survival (OS) curves. * *p* < 0.05, indicating significance.

Patients with pre-treatment *EGFR* T790M mutation had a significantly shorter BMFS compared to those without (*p* = 0.001) (Figure 2A). When the patients were further stratified into subgroups according to their *EGFR*-activating mutations, those with exon 19 deletions combined with T790M mutations had the shortest BMFS, and those with L858R mutations only had the longest BMFS (Figure 2B). On the other hand, patients with *EGFR*-activating mutations detected in their baseline plasma were significantly associated with larger primary tumor sizes (*p* = 0.004), higher node stages (*p* = 0.04), and the development of bone metastasis (*p* = 0.002) (Table 3 and Supplemental Table S1), along with shorter PFS (12.9 months vs. 17.7 months, *p* = 0.02) (Figure 3A) and OS (24.0 months vs. 39.4 months, *p* = 0.02) (Figure 3B), compared to those without *EGFR*-activating mutations detected.

### 2.4. Detection of Tissue and Plasma EGFR Mutations at Disease Progression Using the ddPCR Platform

While all the 79 patients with tumor recurrence had plasma samples available for analysis, rebiopsy was successfully performed in only 35 patients. The plasma and tumor tissue samples at disease progression were subjected to *EGFR* mutation analysis using the ddPCR platform. As shown in Table 4, 57.0% (45/79) of the plasma samples were positive for *EGFR* mutations, including 17 (21.5%; 17/79) L858R mutations, 9 (11.4%; 9/79) L858R combined with T790M mutations, 7 (8.9%; 7/79) exon 19 deletions and 12 (15.2%; 12/79) exon 19 deletions combined with T790M mutations. In the tumor rebiopsy, 94.2% (33/35) were positive for *EGFR* mutations, including 8 (22.9%; 8/35) L858R mutations, 6 (17.1%; 6/35) L858R combined with T790M mutations, 10 (28.6%; 10/35) exon 19 deletions, 8 (22.9%; 8/35) exon 19 deletions combined with T790M mutations and 1 (2.9%; 1/35) T790M mutation only. Overall, acquired *EGFR* T790M mutation was found in 26.6% (21/79) of plasma samples and 42.9% (15/35) of rebiopsy samples. Among the 35 patients who had paired tissue and plasma results at disease progression, the concordance rate for *EGFR* T790M mutation was 71.4% (25/35), with 15.0% (3/20) of the patients showing negative in the tissue testing but positive in the plasma testing (Table 5). The FA of *EGFR*-activating and T790M mutations from plasma and tumor tissues at baseline and at disease progression is shown in Supplemental Figure S2. The FA of T790M detected from plasma or tumor tissues at disease progression was significantly higher than that at baseline; however, the FAs of L858R and exon 19 deletions in plasma or tumor tissues have no statistical difference between baseline and disease progression. Moreover, the presence of pre-treatment *EGFR* T790M mutation in the baseline tissue was not significantly associated with the presence of pre-treatment *EGFR* T790M mutation in the baseline plasma or the emergence of acquired *EGFR* T790M mutation in plasma or rebiopsy tumor tissues at disease progression (Table 6).

## 3. Discussion

Accurately identifying tumors that harbor *EGFR*-activating as well as T790M resistance mutations is critical for the precision management of NSCLC [26]. In this study, through the analyses of *EGFR* mutation status using ddPCR performed in paired tumor and plasma samples obtained from 137 patients with advanced NSCLC prior to their EGFR-TKI treatment and at disease progression, we demonstrated that (1) baseline plasma *EGFR*-activating mutation status can be used as a predictive marker for EGFR-TKI therapy; (2) *EGFR* T790M mutation pre-existing as a minor subpopulation prior to EGFR-TKI treatment is not associated with the emergence of acquired resistance at disease progression; (3) Tissue testing for *EGFR* T790M mutation at disease progression cannot be used as a stand-alone assay, and the analysis of plasma should also be used to more precisely identify patients who might benefit from the subsequent third-generation EGFR-TKI treatment.

By using ddPCR, we found that plasma *EGFR*-activating mutations were detected in 65% (89/137) of patients prior to EGFR-TKI treatment. The positivity of *EGFR*-activating mutation in baseline plasma has been addressed in several previous studies, with a range from 20% to 73% [27]. The variability among studies may result from the differences in the assay sensitivity and disease stages of the enrolled patients. Our results showed that patients with detectable baseline plasma *EGFR*-activating mutations had a shorter PFS and OS compared to those without, suggesting that the absence of detectable *EGFR*-activating mutations in baseline plasma might be used as an indicator for low distant spreading activities and low systemic tumor burden in NSCLC. Our observation of an improved PFS and/or OS to EGFR-TKIs treatment in patients without *EGFR*-activating mutations detected in baseline plasma is also consistent with the previous findings [28,29,30].

Baseline plasma *EGFR* mutation testing is now recommended in the College of American Pathologists (CAP)/International Association for the Study of Lung Cancer (IASLC)/Association for Molecular Pathology (AMP) guidelines for the molecular testing of patients with NSCLC as an alternative for a diagnostic tissue biopsy in cases with insufficient tumor tissue specimens or where tissue specimens are not obtainable; however, its prognostic value for predicting EGFR-TKI outcomes, which has been demonstrated in previous studies, has not yet been applied to clinical practice [31]. One major obstacle for translating plasma *EGFR* mutation testing into routine clinical practice is the lack of standardization of methods for assessing tumor mutations. Therefore, the further evaluation of larger groups of NSCLC patients in prospective studies with respect to diagnosis and prognosis may be warranted for the wide implementation of liquid biopsy.

The seminal mechanism of acquired resistance to first- or second-generation EGFR-TKIs is known to be the emergence of *EGFR* T790M mutation. It is not clear whether *EGFR* T790M mutation in NSCLC patients who have relapsed from first- or second-generation EGFR-TKIs treatment is acquired during disease progression or develops from the pre-existing *EGFR* T790M clones in treatment-naïve patients as a minor population. The presence of pre-treatment *EGFR* T790M mutation in NSCLC has been discovered in several studies using highly sensitive detection methods, such as mass spectrometry, the scorpion amplification refractory mutation system, colony hybridization assays, etc.; the reported prevalence ranges from 20 to 80% [32,33,34,35].

In the present study, *EGFR* T790M mutation in 20.4% (28/137) of the EGFR-mutated, treatment-naïve NSCLC tumors was only detected by using the ddPCR platform but not the cobas EGFR mutation Test, and the FA of *EGFR* T790M is lower than that of *EGFR*-activating mutations in baseline tissue and plasma samples, showing that *EGFR* T790M pre-existing as a minor subpopulation in treatment-naïve, EGFR-mutated NSCLC has undergone clonal expansion in response to the selection pressure by the first- or second-generation EGFR-TKIs treatment. However, through analyses of *EGFR* mutations in plasma and tissue at baseline and disease progression, we found that the presence of *EGFR* T790M mutation at baseline was not statistically associated with the emergence of acquired *EGFR* T790M resistance at disease progression. One possible explanation is that the intratumoral heterogeneity of *EGFR* T790M mutation in tumor tissues results in sampling bias during the tumor biopsy procedures, which contributes to underestimating the incidence of this mutation and leads to the lack of statistical differences found in the present study. Indeed, a study evaluating *EGFR* T790M mutation in the sequential rebiopsies, along with the course of first-generation EGFR-TKI treatment, found that some patients who were *EGFR* T790M-positive at the first post-TKI biopsy became *EGFR* T790M-negative in later post-TKI rebiopsies, and vice versa, which is suggestive of the intratumoral heterogeneity of the mutation [36]. Moreover, the involvement of other molecular changes in the resistance mechanisms may also contribute to the results. In the present study, the concordance rate for *EGFR* T790M mutation was only 71.4% (25/35) between paired tissue and plasma samples at disease progression, with some being *EGFR* T790M-positive in the plasma and negative in the tissue, and vice versa. These findings indicate that tissue or plasma testing for *EGFR* T790M mutation should not be used as a stand-alone assay for selecting patients that are eligible for the subsequent osimertinib treatment, and the repeat or combined testing should be considered.

Our findings that pre-treatment *EGFR* T790M mutation was significantly associated with brain metastasis in patients with EGFR-mutated tumors receiving first- or second-generation EGFR-TKIs suggested that therapeutically targeting the pre-existing minor subpopulation harboring T790M mutation may have the advantage of preventing the development of brain metastasis in NSCLC. In the FLAURA trial, an analysis of a subset of treatment-naïve patients with EGFR-mutated advanced NSCLC and CNS metastases showed that the PFS was longer for patients receiving osimertinib compared to those receiving either gefitinib or erlotinib (15.2 versus 9.6 months; HR 0.47, 95% CI 0.30–0.74), indicating that osimertinib has a higher CNS efficacy in patients with untreated EGFR-mutated NSCLC [14]. Ballard et al. has further validated the CNS activity of osimertinib in a mouse model [37]. The findings in our current study of an association between pre-treatment *EGFR* T790M and brain metastasis might also support the use of osimertinib as a front-line EGFR-TKI treatment in preventing CNS progression.

The prognostic value of pre-treatment *EGFR* T790M mutation in advanced NSCLC patients treated with TKIs remains inconclusive. Some studies showed that patients with detectable *EGFR* T790M mutation had a shorter PFS, but this had no impact on OS, while others found that the presence of pre-treatment *EGFR* T790M mutation indicated favorable outcomes in advanced NSCLC patients treated with EGFR-TKIs [32,33,34,35]. In our study, we found no significant difference in patient survival between those with and without pre-treatment *EGFR* T790M. Possible explanations for the discordance include the differences in assay methodologies, with different sensitivities used among studies, the source of tumor samples (e.g., surgical resection or biopsies) used for analysis and the first- or second-generation EGFR TKIs chosen for treatment. Noteworthily, most patients enrolled in other studies were treated with first-generation EGFR-TKIs, such as gefitinib or erlotinib, but, in our present study, approximately 50% of patients were treated with the second-generation EGFR-TKI afatinib as the first-line therapy. In the phase IIB LUX-Lung 7 trial comparing afatinib and gefitinib as the first-line treatment for EGFR-mutated NSCLC patients, the researchers demonstrated that afatinib significantly improved PFS and time-to-treatment failure compared with gefitinib [38]. In addition, afatinib has been shown to inhibit the growth of gefitinib-resistant lung cancer cells harboring low levels of *EGFR* T790M mutation, but not those with high levels of *EGFR* T790M mutation [25]. Indeed, in this study, we did find that the frequency of developing acquired *EGFR* T790M mutation in patients treated with gefitinib or erlotinib was higher than those treated with afatinib (32.6% vs. 19.4%). In addition, our results showed that patients with detectable baseline *EGFR*-activating mutations are significantly associated with bone metastasis. Previous studies have found that the incidence of *EGFR*-activating mutations in bone metastasis is higher than that in primary adenocarcinomas or metastases to other organs. A study by Furugaki et al. reported that the inhibition of EGFR signaling by erlotinib prevents the tumor-induced osteolytic invasion of NCI-H292 cell lines [39]. These findings suggest that the activation of EGFR signaling promotes the osteolytic invasion and metastasis of tumor cells and provides supportive evidence to our result that baseline plasma *EGFR*-activating mutation status might serve as a predictive marker for the development of bone metastasis in NSCLC.

There were limitations in our current study. First, it was a single-center study with a small sample size due to the difficulty in longitudinally collecting the tissue and plasma samples in advanced NSCLC patients prior to treatment and at disease progression, and further investigation in a larger cohort may be required to confirm the findings. Second, the assay sensitivity and specificity towards *EGFR* mutation testing using the ddPCR platform cannot be accurately evaluated in this study because of a lack of enrolled *EGFR* mutation-negative patients. Third, patients enrolled in this study were at advanced stages, and their tumor tissues were obtained via tumor biopsy. The intratumor heterogeneity of *EGFR* T790M mutation may lead to an underestimation of the prevalence due to sampling bias, which may, in turn, influence its clinical impact.

In conclusion, our study demonstrated the significance of using the ultra-sensitive ddPCR platform for the dynamic assessment of tissue and plasma *EGFR* mutation status at baseline and disease progression in EGFR mutant NSCLC treated with EGFR-TKIs. In addition to evaluating the EGFR-TKI treatment efficacy and the emergence of resistance mechanisms, the use of the ddPCR method for *EGFR* mutation detection is proven to be valuable in predicting tumor metastasis and patient outcomes.

## 4. Materials and Methods

### 4.1. Study Population

**Figure 4:**
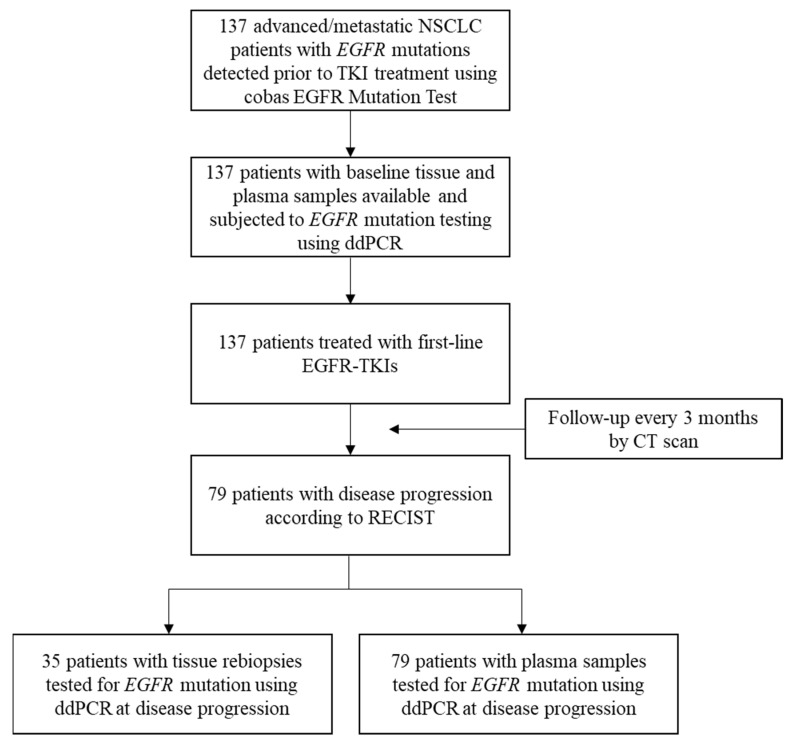
Flow diagram of the study population. NSCLC, non-small cell lung cancer; ddPCR, droplet digital polymerase chain reaction; CT, computed tomography.

This was a single-center prospective observational study. Patients who were pathologically diagnosed with lung adenocarcinoma, were positive for *EGFR*-activating mutations determined by the routine cobas EGFR Mutation Test (v1 was used before 31 March 2017, and v2 was applied thereafter; Roche Molecular Systems, Inc.), had inoperable stage IIIB or IV diseases according to the 7th edition of the American Joint Committee for Cancer staging system [26] and planned to receive EGFR-TKIs as their first-line therapy with either gefitinib, erlotinib or afatinib were enrolled. Gefitinib, erlotinib and afatinib are the only first-line EGFR-TKIs that have been reimbursed by the Taiwan National Health Insurance scheme for advanced *EGFR* mutation-positive lung adenocarcinoma; therefore, patients with osimertinib as a first-line treatment were not available in this study. Patients were excluded if they had another active malignancy or any prior treatment that could influence the tumor burden. All enrolled patients were subjected to blood collection according to standard procedures at baseline (within 7 days before the first dose of EGFR-TKI) and at the time of disease progression. Treatment response was evaluated every 3 months with computed tomography (CT) by specialized radiologists, according to the Response Evaluation Criteria in Solid Tumors version 1.1 [40]. From June 2015 to July 2018, a total of 137 lung adenocarcinoma patients at Taipei Veterans General Hospital (Taipei city, Taiwan) were enrolled in this study (Figure 4). The prior-to-treatment (at baseline) and rebiopsy (at disease progression) tumor tissues and plasma samples were analyzed for *EGFR* mutation status using ddPCR. Clinical characteristics, including the patients’ age, gender, smoking history, distant organ metastasis, first-line EGFR-TKIs, *EGFR* mutation status, date of initial diagnosis, treatment start date, time to disease progression, date of death or last follow-up, etc., were collected. Never-smokers were defined as patients who had never smoked cigarettes, whereas smokers were defined as those who were current or former smokers. The study protocol was approved by the institutional review board (IRB) of Taipei Veterans General Hospital.

### 4.2. DNA Isolation from Plasma and Formalin-Fixed Paraffin-Embedded Tissues

For cfDNA isolation, 10 mL of peripheral blood was collected in a K2 EDTA tube and was processed within 4 h. The tubes were centrifuged at 1600× *g* for 15 min, and cfDNA was isolated using the cobas cfDNA Sample Preparation Kit (Roche Molecular Systems, Inc., Pleasanton, CA, USA) according to the manufacturer’s protocol. For the genomic DNA isolation from tumor tissues, formalin-fixed paraffin-embedded (FFPE) tissues were stained with Hematoxylin & Eosin (H&E) and reviewed by pathologists to select the tumor areas. The selected areas were then manually macrodissected from the corresponding ones in the consecutive tissue sections and, after deparaffinization, were subjected to genomic DNA extraction using the cobas DNA Sample Preparation Kit (Roche Molecular Systems, Inc., Pleasanton, CA, USA) according to the manufacturer’s instructions.

### 4.3. EGFR Mutation Analysis Using Droplet Digital PCR

This assay was performed on a QX200 Droplet Digital PCR system (Bio-Rad Laboratories, Inc., Hercules, CA, USA), in which a PCR sample is partitioned into approximately 20,000 droplets. The mean of the accepted droplets in the samples of this study was 14,725 with a standard deviation of 1609 droplets. After amplification, each droplet is scored as positive or negative via fluorescence detection of the target sequence. Poisson statistical analysis is used for the absolute quantification of the target sequence. For *EGFR* mutation analysis, the ddPCR Mutation Detection Assay Kits for the analysis of *EGFR* T790M Mutation and *EGFR* L858R Mutation and the ddPCR *EGFR* Exon 19 Deletions Screening Kit purchased from Bio-Rad Laboratories, Inc., (Hercules, CA, USA) were applied according to the manufacturer’s instructions. The emulsified samples were then transferred to 96-well plates for the PCR reaction. The PCR condition used was as follows: initial enzyme activation for 10 min at 95 °C, 40 cycles of 30 sec denaturation at 94 °C, annealing/extension for 1 min at 55 °C and then enzyme deactivation at 98 °C for 10 min. Subsequently, the PCR products were loaded into the QX200 droplet reader for analysis using QuantaSoft Software version 1.7.4.0917 (Bio-Rad Laboratories, Inc., Hercules, CA, USA). A series of experimental controls were also performed in each assay, including a no template control (NTC) for monitoring environmental contamination, a negative control (the *EGFR* wild-type reference standard purchased from Horizon Discovery Group PLC, Cambridge, UK) for the evaluation of the false positive rates and a positive control (the *EGFR* mutation-specific reference standard purchased from Horizon Discovery Group PLC, Cambridge, UK) for the confirmation of the assay performance and the determination of the threshold value. In ddPCR analysis, the limit of blank (LOB) is defined as a finite number of false events of mutant droplets detected in each assay [41]. By evaluating a total of 20 normal samples, including 10 non-tumor lung tissue and 10 normal plasma samples, the LOB was determined by fitting a Poisson model to the false positive frequency distribution for each target and evaluating the 95% one-tailed upper limit of the model distribution. The number of false positive droplet events ranged from 0 to 2 for T790M and was 0 for L858R or exon 19 deletions. Therefore, the positive threshold for each target was set to at least three positive droplets detected in the assay. According to the instructions for the ddPCR Mutation Detection Assay Kits and ddPCR *EGFR* Exon 19 Deletions Screening Kit provided by the manufacturer (Bio-Rad Laboratories, Inc., Hercules, CA, USA), the limit of detection (LoD) for L858R and T790M was claimed to be 0.1%, and that for exon 19 deletions was 0.5%. To further verify the LoD, *EGFR* mutation-specific reference standards (Horizon Discovery Group PLC, Cambridge, UK) were serially diluted with the *EGFR* wild-type reference standard (Horizon Discovery Group PLC, Cambridge, UK) to the specified variant allele frequencies (VAFs) from 50% to 0.1%. The input DNA with varying proportions of mutant DNA mixed into wild-type DNA was subjected to ddPCR analysis. All reactions were repeated three times at each VAF. The minimum VAF that can be reliably detected (coefficient of variation (CV) < 30%) was 0.1% for L858R, exon 19 deletions and T790M (Supplemental Figure S1). Therefore, in this study, the LoD for L858R and T790M was defined as 0.1%, and for exon 19 deletions, it was 0.5%. Samples with at least three positive droplets detected and with a fractional abundance (FA; %) ≥ LoD were considered as mutation-positive cases. All specimens with pre-treatment *EGFR* T790M detected were independently re-tested to confirm the obtained positive results.

### 4.4. ddPCR Data Analysis

The ddPCR data were analyzed using QuantaSoft Software version 1.7.4.0917 (Bio-Rad Laboratories, Inc., Hercules, CA, USA), by which the numbers of positive and negative droplets for each fluorophore in each sample were measured. It then fits the fraction of positive droplets to a Poisson algorithm to determine the starting concentration of the target DNA molecule in units of copies/µL input. The absolute quantification mode used for the concentration calculation of target molecules is based on the following formula:

Concentration (copies/µL) = −ln (N–neg/N–total)/V–droplet

[N–neg = the number of total negative droplets in a well; N–total = the number of total droplets in a well; V–droplet = the volume of a droplet]

Then, Fractional Abundance (FA), referring to the proportion of mutant allele frequencies by QuantaSoft, is auto-calculated by the software with the formula:

FA = Concentration of mutant allele/(Concentration of mutant allele + Concentration of wild-type allele)

### 4.5. Statistical Analysis

The concordance rates between the tissue and the plasma and between the different assay platforms were calculated as the number of positive and negative samples out of the total number of tested samples. The Kaplan–Meier method was used to analyze the progression-free survival (PFS), overall survival (OS) and brain metastasis-free survival (BMFS), and the log-rank test was used to compare the survival times between groups and to calculate the hazard ratio with a 95% confidence interval. The Chi-square test or Fisher’s exact test was used to analyze the associations between the *EGFR* mutation status and clinical characteristics of patients. The association between clinical factors and the pre-treatment *EGFR* T790M mutation or plasma *EGFR*-activating mutations was also examined using multivariate regression analyses. A *p* value < 0.05 was considered to indicate a statistically significant difference. All statistical analyses were conducted using SAS Statistics v.6.1 and GraphPad Prism v.8.0.

## Figures and Tables

**Table 1:** Comparison of *EGFR* mutation status determined from baseline tumor tissues using the cobas EGFR Mutation Test and the ddPCR platform (N = 137).

	Cobas EGFR Mutation Test		ddPCR	
	No.	(%)	No.	(%)
L858R	77	(56.2)	61	(44.5)
L858R/T790M	0	(0.0)	16	(11.7)
E19D	60	(43.8)	48	(35.0)
E19D/T790M	0	(0.0)	12	(8.8)
Total	137	(100.0)	137	(100.0)

The concordance rate for overall *EGFR* mutations is 79.6% (109/137). The concordance rate for activating *EGFR* mutations alone is 100% (137/137). All percentages have been rounded off. Column totals differing from the numbers added are a result of rounding errors. Abbreviations: ddPCR, droplet digital polymerase chain reaction; E19D, exon 19 deletions.

**Table 2:** Comparison of *EGFR* mutation status determined from baseline tumor tissues and plasma samples using the ddPCR platform (N = 137).

	Tumor Tissues									
	L858R		L858R/T790M		E19D		E19D/T790M		Total	
Plasma	No.	(%)	No.	(%)	No.	(%)	No.	(%)	No.	(%)
L858R	37	(27.0)	10	(7.3)	0	(0.0)	0	(0.0)	47	(34.3)
L858R/T790M	1	(0.7)	1	(0.7)	0	(0.0)	0	(0.0)	2	(1.5)
E19D	0	(0.0)	0	(0.0)	32	(23.4)	6	(4.4)	38	(27.7)
E19D/T790M	0	(0.0)	0	(0.0)	1	(0.7)	1	(0.7)	2	(1.5)
Not detected	23	(16.8)	5	(3.6)	15	(10.9)	5	(3.6)	48	(35.0)
Total	61	(44.5)	16	(11.7)	48	(35.0)	12	(8.8)	137	(100.0)

The concordance rate for overall *EGFR* mutations is 51.8% (71/137). The concordance rate for activating *EGFR* mutations alone is 65.0% (89/137). All percentages have been rounded off. Column totals differing from the numbers added are a result of rounding errors. Abbreviations: ddPCR, droplet digital polymerase chain reaction; E19D, exon 19 deletions.

**Table 3:** Patient characteristics stratified by tissue *EGFR* T790M or plasma *EGFR*-activating mutation status at baseline (N = 137).

	All		Tissue					Plasma				
			T790M+		T790M−		*p* Value	*EGFR* m+		*EGFR* m−		*p* Value
	No.	(%)	No.	(%)	No.	(%)		No.	(%)	No.	(%)	
Number of cases	137	(100.0)	28	(20.4)	109	(79.6)		89	(65.0)	48	(35.0)	
Stage ^a^							0.99					0.54
IIIB	2	(1.5)	0	(0.0)	2	(1.8)		2	(2.2)	0	(0.0)	
IV	135	(98.5)	28	(100.0)	107	(98.2)		87	(97.8)	48	(100.0)	
Sex							0.82					0.68
Male	61	(44.5)	13	(46.4)	48	(44.0)		40	(44.9)	21	(43.8)	
Female	76	(55.5)	15	(53.6)	61	(56.0)		49	(55.1)	27	(56.2)	
Age												
Mean	64.3		63.0		64.7		0.49	63.7		65.5		0.37
Range	37.3–90.7		37.3–83.2		45.4–90.7			37.3–90.7		45.4–90.6		
Smoking history							0.20					0.51
Never-smoker	101	(73.7)	18	(64.3)	83	(76.1)		64	(71.9)	37	(77.1)	
Smoker	36	(26.3)	10	(35.7)	26	(23.9)		25	(28.1)	11	(22.9)	
First-line therapy							0.33					0.11
Afatinib	74	(54.0)	16	(57.1)	58	(53.2)		51	(57.3)	23	(47.9)	
Erlotinib	41	(29.9)	10	(35.7)	31	(28.4)		28	(31.5)	13	(27.1)	
Gefitinib	22	(16.1)	2	(7.1)	20	(18.3)		10	(11.2)	12	(25.0)	
Primary tumor size, mm							0.30					0.004 ^b^
Mean	40.0		43.9		39.0			42.9		34.2		
Range	12–159		14–76		12–159			12–159		16–76		
Tumor category							0.68					0.69
T1	25	(18.2)	3	(10.7)	22	(20.2)		16	(18.0)	9	(18.8)	
T2	50	(36.5)	12	(42.9)	38	(34.9)		33	(37.0)	17	(35.4)	
T3	20	(14.6)	4	(14.3)	16	(14.7)		15	(16.9)	5	(10.4)	
T4	42	(30.7)	9	(32.1)	33	(30.3)		25	(28.1)	17	(35.4)	
Nodal status							0.42					0.04 ^b^
N0	43	(31.4)	11	(39.3)	32	(29.4)		23	(25.9)	20	(41.7)	
N1	15	(10.9)	3	(10.7)	12	(11.0)		10	(11.2)	5	(10.4)	
N2	43	(31.4)	10	(35.7)	33	(30.3)		26	(29.2)	17	(35.4)	
N3	36	(26.3)	4	(14.3)	32	(29.4)		30	(33.7)	6	(12.5)	
Metastasis status ^a^							0.54					0.05
M0	3	(2.3)	0	(0.0)	3	(2.8)		2	(2.2)	1	(2.1)	
M1a	35	(25.5)	5	(17.9)	30	(27.5)		16	(18.0)	19	(39.5)	
M1b	75	(54.7)	18	(64.3)	57	(52.3)		54	(60.7)	21	(43.8)	
M1c	24	(17.5)	5	(17.9)	19	(17.4)		17	(19.1)	7	(14.6)	
*EGFR* mutation							0.91					N/A
L858R	77	(56.2)	16	(57.1)	61	(56.0)		49	(55.1)	0	(0.0)	
E19D	60	(43.8)	12	(42.9)	48	(44.0)		40	(44.9)	0	(0.0)	
Brain-Metastasis							0.005 ^b^					0.34
Yes	56	(40.9)	18	(64.3)	38	(34.9)		39	(43.8)	17	(35.4)	
No	81	(59.1)	10	(35.7)	71	(65.1)		50	(56.2)	31	(64.6)	
Bone-Metastasis							0.84					0.002 ^b^
Yes	71	(51.8)	15	(53.6)	56	(51.4)		55	(61.8)	16	(33.3)	
No	66	(48.2)	13	(46.4)	53	(48.6)		34	(38.2)	32	(66.7)	
Pleura-Metastasis							0.15					0.65
Yes	34	(24.8)	4	(14.3)	30	(27.5)		21	(23.6)	13	(27.1)	
No	103	(75.2)	24	(85.7)	79	(72.5)		68	(76.4)	35	(72.9)	
Liver-Metastasis ^a^							0.23					0.14
Yes	13	(9.5)	1	(3.6)	12	(11.0)		11	(12.4)	2	(4.2)	
No	124	(90.5)	27	(96.4)	97	(89.0)		78	(87.6)	46	(95.8)	

Abbreviations: E19D, exon 19 deletions; N/A: not applicable. All percentages have been rounded off. Column totals differing from 100 are a result of rounding errors. ^a^ Fisher’s exact test was applied due to an expected value of less than five. ^b^ *p* < 0.05, indicating significance.

**Table 4:** *EGFR* mutation status determined from the plasma and tumor rebiopsy samples using the ddPCR platform at disease progression.

	Plasma		Tumor Rebiopsy	
	No.	%	No.	%
No. of patients	79	100.0	35	100.0
L858R	17	21.5	8	22.9
L858R/T790M	9	11.4	6	17.1
E19D	7	8.9	10	28.6
E19D/T790M	12	15.2	8	22.9
T790M	0	0.0	1	2.9
No variation detected	34	43.0	2	5.7

All percentages have been rounded off. Column totals differing from 100 are a result of rounding errors. Abbreviations: ddPCR, droplet digital polymerase chain reaction; E19D, exon 19 deletions.

**Table 5:** Comparison of *EGFR* T790M mutation status determined from the plasma and tumor rebiopsy samples at disease progression (N = 35).

	Tumor Rebiopsy					
	T790M+		T790M−		Total	
	No.	(%)	No.	(%)	No.	(%)
Plasma						
T790M+	8	(22.9)	3	(8.5)	11	(31.4)
T790M−	7	(20.0)	17	(48.6)	24	(68.6)
Total	15	(42.9)	20	(57.1)	35	(100.0)

All percentages have been rounded off.

**Table 6:** Summary of *EGFR* T790M mutation status in tissue and plasma tested at baseline and disease progression (N = 137).

	Tissue at Baseline				
	T790M+		T790M−		*p* Value
	No.	(%)	No.	(%)	
No. of patients	28	(100.0)	109	(100.0)	
Plasma at baseline ^a^					0.19
T790M+	2	(7.1)	2	(1.8)	
T790M−	26	(92.9)	107	(98.2)	
Plasma at progression					0.36
T790M+	6	(21.4)	15	(13.8)	
T790M−	11	(39.3)	47	(43.1)	
N/A	11	(39.3)	47	(43.1)	
Tumor Rebiopsy at progression ^a^					0.45
T790M+	5	(17.9)	10	(9.2)	
T790M−	4	(14.3)	16	(14.6)	
N/A	19	(67.8)	83	(76.2)	

N/A: Not available. All percentages have been rounded off. ^a^ Fisher’s exact test was applied due to an expected value of less than five.

## Data Availability

The data presented in this study are available on request from the corresponding author.

## Supplementary Materials

The following supporting information can be downloaded at: https://www.mdpi.com/article/10.3390/ijms231911353/s1.
