# Supplementary material for: Dynamic Assessment of Tissue and Plasma EGFR-Activating and T790M Mutations with Droplet Digital PCR Assays for Monitoring Response and Resistance in Non-Small Cell Lung Cancers Treated with EGFR-TKIs
Source: Int J Mol Sci. 2022 Sep 26;23(19):11353. doi: 10.3390/ijms231911353 (PMC9569685; doi:10.3390/ijms231911353)
Supplement: Supplementary file 1 [file ijms-23-11353-s001.zip › 20220921 ddPCR IJMS Supplemental Figures FINAL.pptx]

## Slide 1
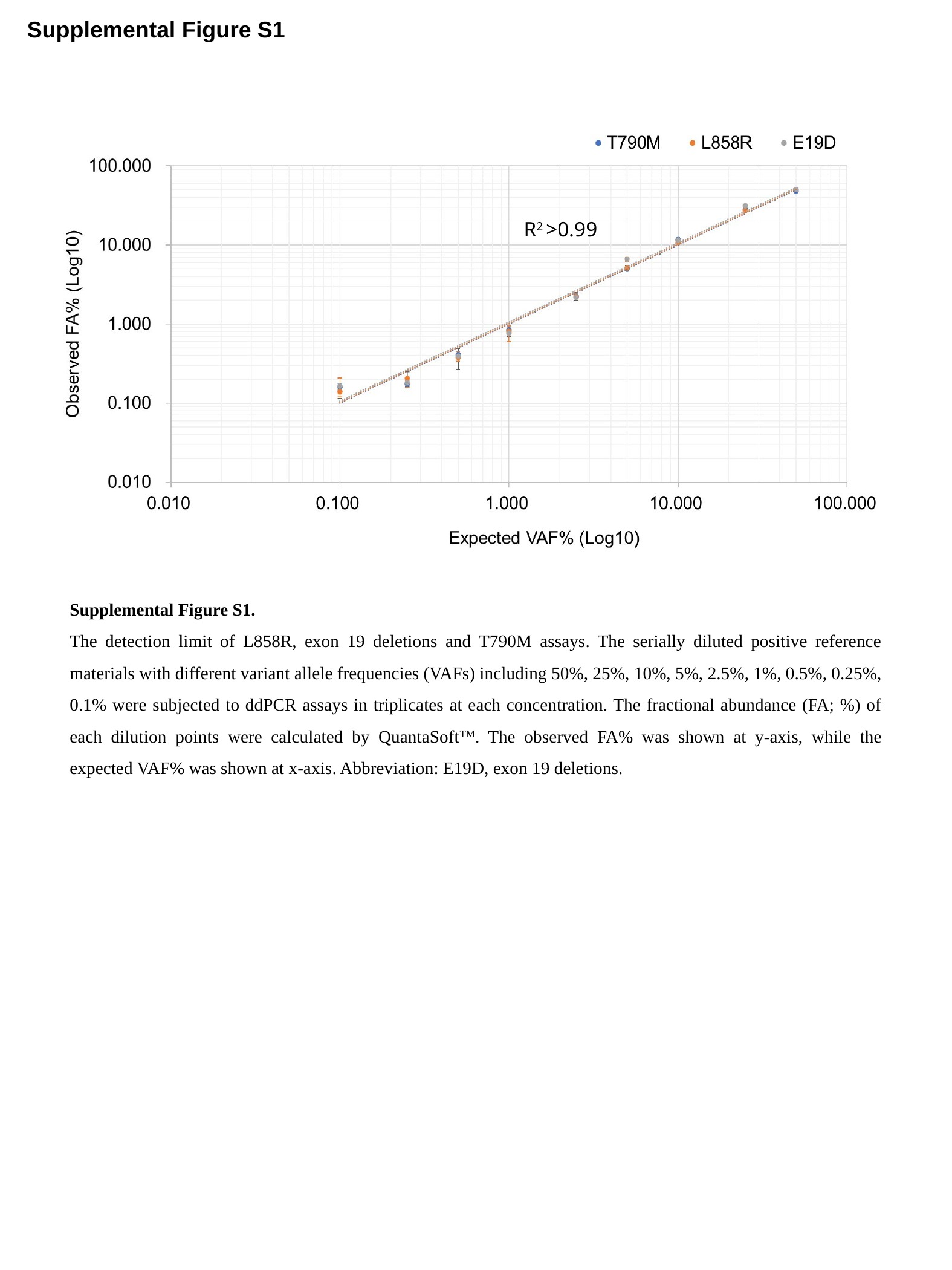

Supplemental Figure S1
R2 >0.99
Supplemental Figure S1.
The detection limit of L858R, exon 19 deletions and T790M assays. The serially diluted positive reference materials with different variant allele frequencies (VAFs) including 50%, 25%, 10%, 5%, 2.5%, 1%, 0.5%, 0.25%, 0.1% were subjected to ddPCR assays in triplicates at each concentration. The fractional abundance (FA; %) of each dilution points were calculated by QuantaSoftTM. The observed FA% was shown at y-axis, while the expected VAF% was shown at x-axis. Abbreviation: E19D, exon 19 deletions.

## Slide 2
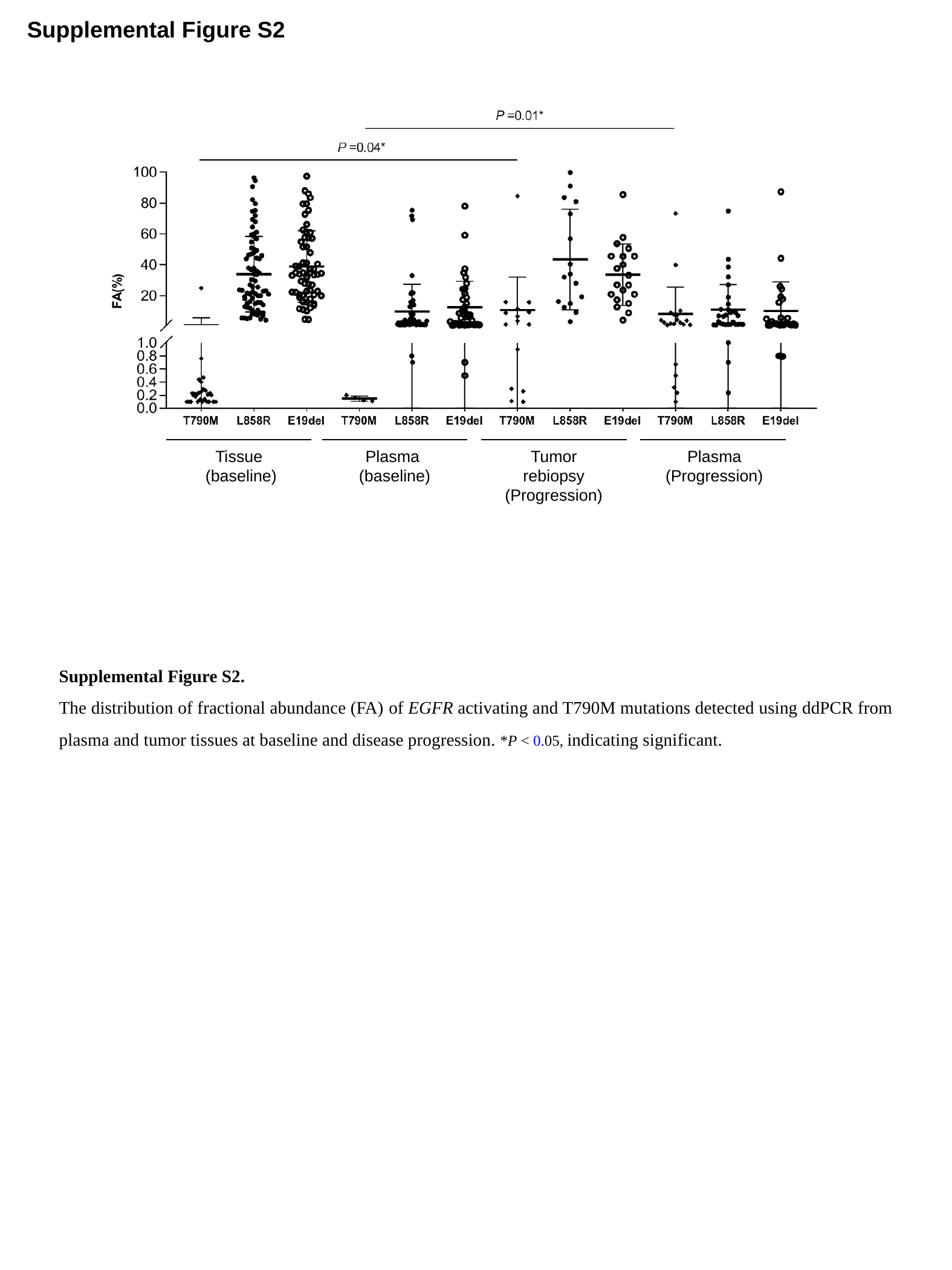

Supplemental Figure S2
Tissue
 (baseline)
Plasma
(baseline)
Tumor rebiopsy (Progression)
Plasma (Progression)
Supplemental Figure S2.
The distribution of fractional abundance (FA) of EGFR activating and T790M mutations detected using ddPCR from plasma and tumor tissues at baseline and disease progression. *P < 0.05, indicating significant.
