# Supplementary material for: Dynamic Assessment of Tissue and Plasma EGFR-Activating and T790M Mutations with Droplet Digital PCR Assays for Monitoring Response and Resistance in Non-Small Cell Lung Cancers Treated with EGFR-TKIs
Source: Int J Mol Sci. 2022 Sep 26;23(19):11353. doi: 10.3390/ijms231911353 (PMC9569685; doi:10.3390/ijms231911353)
Supplement: Supplementary file 1 [file ijms-23-11353-s001.zip › 20220921 ddPCR IJMS Supplemental Tables FINAL.pdf]

Supplemental Table S1. Multivariate analysis of clinicopathological parameters associating with the detection of tissue *EGFR* T790M or plasma *EGFR* activating mutations at baseline.

Factors	Number of cases	Tissue T790M +			Plasma *EGFR*m+		
		Multivariate analysis			Multivariate analysis		
		Odds ratio	95% CI	*P*	Odds ratio	95% CI	*P*
Sex							
Male	61	1			1		
Female	76	1.27	(0.30-5.45)	*0.75*	1.83	(0.58-5.80)	*0.31*
Age							
≤ 65 y/o	58	1			1		
> 65 y/o	79	0.59	(0.20-1.78)	*0.35*	1.22	(0.47-3.13)	*0.68*
Smoking history							
Never smoker	101	1			1		
Smoker	36	3.08	(0.69-13.88)	*0.14*	1.82	(0.51-6.41)	*0.35*
First-line therapy							
Afatinib	74	1			1		
Erlotinib	41	1.03	(0.33-3.19)	*0.96*	0.8	(0.28-2.23)	*0.66*
Gefitinib	22	0.39	(0.06-2.57)	*0.33*	0.25	(0.07-0.93)	*0.04* [a]
Primary tumor size							
> 40 mm	62	1			1		
≤ 40 mm	58	2.69	(0.79-9.15)	*0.11*	3.77	(1.25-11.4)	*0.02* [a]
n.a.	17						
Tumor category							
T1	25	1			1		
T2	50	3.43	(0.66-17.93)	*0.14*	1.45	(0.41-5.15)	*0.57*
T3	20	1.58	(0.20-12.56)	*0.66*	1.43	(0.26-7.72)	*0.68*

T4	42	2.45	(0.43-13.99)	*0.31*	0.81	(0.22-3.02)	*0.76*
Nodal status							
N0	43	1			1		
N1	15	0.28	(0.04-1.78)	*0.18*	1.19	(0.25-5.78)	*0.83*
N2	43	0.37	(0.10-1.33)	*0.13*	1.15	(0.39-3.36)	*0.80*
N3	36	0.16	(0.03-0.79)	*0.03* [a]	4.55	(1.24-16.7)	*0.03* [a]
Metastasis status							
M0	3	1			1		
M1a	35	N/A	N/A	*N/A*	0.89	(0.05-15.3)	*0.94*
M1b	75	N/A	N/A	*N/A*	1.38	(0.07-27.9)	*0.83*
M1c	24	N/A	N/A	*N/A*	1.05	(0.05-24.4)	*0.97*
EGFR mutation							
L858R	77	1			1		
E19del	60	0.95	(0.34-2.68)	*0.92*	N/A	N/A	*N/A*
Brain-Metastasis							
Yes	56	1			1		
No	81	0.19	(0.05-0.67)	*0.01* [a]	1.36	(0.45-4.05)	*0.59*
Bone-Metastasis							
Yes	71	1			1		
No	66	1.08	(0.34-3.47)	*0.90*	0.29	(0.10-0.87)	*0.03* [a]

[a] $P < 0.05$, indicating significant

n.a.: Not available

N/A: Not applicable
